# Race and Concussion: An Emerging Relationship

**DOI:** 10.31486/toj.20.0145

**Published:** 2020

**Authors:** Ryan Wagner, Karie Zach, Yuka Kobayashi, Andrew W. Gottschalk

**Affiliations:** ^1^ Department of Sports Medicine, Medical College of Wisconsin, Milwaukee, WI; ^2^ Department of Family and Sports Medicine, Oregon Health and Science University, Portland, OR; ^3^ Department of Orthopedics, Sports Medicine Institute, Ochsner Clinic Foundation, New Orleans, LA

## CASE

Two 15-year-old football players from opposing teams are involved in a head-to-head collision during a game. One player is African American; the other is Caucasian. They both immediately develop symptoms consistent with a concussion. The Caucasian athlete reports his symptoms to his athletic trainer and is removed from the game. The African American athlete shakes it off and plays the rest of the game with continued symptoms. Why the discrepancy?

## BACKGROUND

Traumatic brain injury (TBI) is a serious public health concern, resulting in significant morbidity and mortality for thousands of people each year.^1^ More than 85% of TBIs are classified as mild and usually result from sport-related impacts.^2,3^ Sport-related concussion (SRC), a mild TBI induced by biomechanical forces, has the potential to cause acute and chronic neuropathologic changes, as well as clinical symptoms such as behavioral changes, cognitive impairment, and sleep disturbance.^4^ The relationships between SRCs and epidemiologic factors (eg, race, socioeconomic status) are beginning to emerge.

## REVIEW OF EVIDENCE

In 2017, the Centers for Disease Control and Prevention (CDC) released an analysis of data from the national Youth Risk Behavior Survey, a biennial survey of 14,765 students in grades 9 to 12.^5^ An increased prevalence of concussions among male students compared to female students was noted. Across all racial and ethnic subgroups, youth athletes who were involved in multiple sports were more likely to report concussions. The CDC recommended focused efforts on educating youth athletes regarding the risks of concussions, regardless of the risk of concussion in a particular sport.^5^

The few studies that delineate the relationship between SRC and racial disparities focus on the adolescent athlete.^6,7^ African Americans comprise the majority of underserved, low-income student athletes in urban communities across the United States. Commonly, their schools lack access to health care professionals, especially licensed athletic trainers, who can provide concussion education and management, neuropsychologic baseline testing, and appropriate recommendations for returning to play after concussion. Illustrating the consequences of the lack of resources, a 2018 study found that African American adolescent athletes exhibited less concussion knowledge and were less likely to recognize concussion symptoms compared to Caucasian athletes.^6^ The study demonstrated that student athletes with access to licensed athletic trainers had greater concussion knowledge than athletes without access to trainers.^6^ Research suggests a greater risk of neurocognitive impairment following SRC in African American athletes.^8^

The finding that African American athletes have less knowledge about head injuries than Caucasian athletes aligns with evidence found in a retrospective, cross-sectional analysis of emergency department (ED) visits from the National Electronic Injury Surveillance System for the period 2008 to 2017. Of the 11,529,994 injuries for pediatric sport-related injuries, 13% were injuries to the head, and 5.4% of those patients were diagnosed with SRC.^9^ African American patients were less likely than Caucasian patients to have ED visits for head injuries or concussions and were less likely to be diagnosed with a concussion during an ED visit.^9^

A paucity of research exists regarding the role of socioeconomic status in SRC outcomes. Zuckerman et al conducted a 3-year retrospective cohort study on the impact of socioeconomic status on SRC outcomes of 282 athletes (middle school through college). Factors—most based on ZIP code—used to define socioeconomic status were cost of living, median income, percentage of college graduates, home ownership, county setting, and insurance status.^7^ The outcomes investigated were symptom duration, days of missed school, and days of missed practices. Results from the study indicated that no socioeconomic factors correlated with duration of symptoms or return to sport; however, athletes with public insurance returned to school quicker than athletes with private insurance.

Zuckerman and colleagues suggested an explanation for this finding. Athletes with private health insurance have a greater probability of receiving care from a concussion specialist who may advocate for cognitive testing and relative rest, leading to an increased number of missed school days. Conversely, athletes with public insurance were unable to receive care from a specialist and therefore returned to school based on their symptoms or recommendations from parents or teachers.^7^ This explanation is reinforced by CDC findings that racial and ethnic minority groups are less likely to receive follow-up care by health care professionals after evaluation in the ED for mild TBIs.^1^

## TAKEAWAY

As research into the relationship between SRC and racial disparities continues, providing concussion education to at-risk populations is crucial. Achieving optimal outcomes requires access to appropriate health care specialists on the sidelines and in the clinic setting. With focused attention from national sport organizations, school administrators, parents, coaches, and medical personnel on barriers in specific populations, the at-risk athlete sustaining an SRC will have a greater chance to successfully return to sport, and more importantly, to the classroom.

## CASE RESOLUTION

The Caucasian athlete recognized and reported his symptoms to the licensed athletic trainer. He was appropriately removed from the game. His symptoms resolved completely in 2 weeks, and he played the rest of the football season. The African American athlete, not aware that he had sustained a concussion, played the remainder of the game with persistent symptoms. He had a complicated and prolonged recovery and was unable to return to football during the season.

## Notes

This article meets the Accreditation Council for Graduate Medical Education and the American Board of Medical Specialties Maintenance of Certification competencies for Patient Care and Medical Knowledge.
